# Multiple insecticide resistance in *Culex quinquefasciatus* populations from Guadeloupe (French West Indies) and associated mechanisms

**DOI:** 10.1371/journal.pone.0199615

**Published:** 2018-06-26

**Authors:** Christelle Delannay, Daniella Goindin, Kevin Kellaou, Cédric Ramdini, Joël Gustave, Anubis Vega-Rúa

**Affiliations:** 1 Laboratory of Medical Entomology, Environment and Health Unit, Institut Pasteur de la Guadeloupe, Les Abymes, Guadeloupe, France; 2 Vector Control Service of Grande–Terre, Regional Health Agency, Airport Zone South Raizet, Les Abymes, Guadeloupe, France; Universidade Federal de Vicosa, BRAZIL

## Abstract

West Nile (WN) virus has been detected in Guadeloupe since 2002. Even if no WN human cases have been detected so far, mosquitoes from *Culex* genus especially *Culex quinquefasciatus* are recognized as potential WN vectors in Guadeloupe. To evaluate the impact of local vector control activities on this mosquito species we assessed the resistance levels of *Cx*. *quinquefasciatus* populations from three different sites from Guadeloupe (Abymes, Saint François and Gourbeyre) to malathion, temephos and deltamethrin. In addition, the frequencies of the L1014F *kdr* and the G119S *ace-1* mutations were established in *Cx*. *quinquefasciatus* populations, as well as the constitutive expressions of five cytochrome P450 genes. Mosquito populations tested displayed high resistance to deltamethrin, moderate resistance to malathion (Abymes, Gourbeyre) and low resistance to temephos (Abymes et Gourbeyre). Molecular analyses revealed high frequencies of both L1014F *kdr* and G119S *ace*-1 mutations in *Cx*. *quinquefasciatus* populations, as well as overexpression of cytochrome P450 genes *CYP9J45*, *CYP9J40* and *CYP6AA7*. Finally, deltamethrin resistance and knock-down rates were strongly correlated with the frequency of the resistant *kdr* and *ace-1* alleles, as well as with *CYP9J40* overexpression. These results should be taken into account to refine the current vector control strategies to prevent the appearance of *Cx*. *quinquefasciatus*-borne diseases in Guadeloupe.

## Introduction

During the past 30 years, the Guadeloupe islands (French West Indies) have been facing arboviral outbreaks with increasing frequency, number of cases and fatalities. The largest dengue outbreak ever described in this territory was reported in 2010 involving around 44,000 dengue-like syndromes [1]. Since then, a Chikungunya epidemic (80,000 suspect cases) hit the Guadeloupian archipelago between 2013 and 2015 [2], shortly followed by a Zika virus epidemic that led to 28,345 suspect cases [3,4], 67 severe neurological complications and 14 foetal neurological pathologies, including microcephaly [5].

As the arboviruses causing dengue (DENV), chikungunya (CHIKV) and Zika (ZIKV) are mainly transmitted in the Caribbean by the urban mosquito *Aedes aegypti* (Linnaeus 1762) [6–9], vector control activities in Guadeloupe focus mainly in this mosquito species. Nevertheless, since 2002 West Nile virus (WNV; Flavivirus) has also been detected in Guadeloupe during several serological investigations performed on horses and chickens [10,11]. WNV is the cause of the most widespread vector-borne disease in the world and one of the largest outbreak of neuroinvasive disease ever observed [12]. WNV circulates in nature via an enzootic cycle between ornithophilic mosquitoes (i.e. *Culex* genus), and susceptible birds [12–14]. In Guadeloupe, no human WNV cases have been reported so far [15], but the possibility of WNV emergence in urban settings remains a real concern. Indeed, the southern house mosquito *Culex quinquefasciatus* (Say 1823) is the most abundant mosquito species in urban environments of French West Indies after *Ae*. *aegypti* [16–18], and is a highly anthropophilic species that have been proven to be competent to transmit WNV in the Americas [19–21].

Vector control activities in Guadeloupe have been historically carried out by a Vector Control Agency from the French Ministry of local Health (Agence Régionale de Santé; ARS). The ARS ensures larval reduction mainly through the mechanical elimination of breeding sites, the use of larvivorous fish *Poecilia reticulata* and larvicide treatments, while adult densities reduction is also directly conducted by spraying chemical adulticides outdoor and indoor [22]. The insecticides commonly used in the past have been the larvicide temephos and the adulticides deltamethrin and malathion [23,24]. However, temephos and malathion have been withdrawn from the list of vector control insecticides following European Union recommendations in 2009 and 2010 respectively, and have been since then completely replaced by the biological larvicide *Bacillus thuringiensis* var. *israelensis* (Bti) and the deltamethrin [22,25].

The deployment of insecticides to control mosquito vectors has allowed the selection of resistance around the world [26,27]. The two main insecticide resistance mechanisms in mosquitoes are the enzymatic detoxification (also known as metabolic resistance) and the modification of the molecular site targeted by the neurotoxic insecticide chemical (also known as target-site modification) [28,29]. The major classes of enzymes involved in metabolic resistance are esterases, glutathione-S-transferases and cytochrome P450 oxidases [28,29]. All these classes are involved in resistance to different insecticides families, but cytochrome P450 oxidases play a major role in resistance to pyrethroids such as deltamethrin and permethrin [29–31]. Concerning the target-site modifications, mutations in voltage-gated sodium channels (VGSC) have been found to confer resistance to pyrethroids, while mutations in the synaptic acetylcholinesterase *ace-1* gene confer resistance to organophosphates and carbamates [32,33]. *Ae*. *aegypti* populations from Guadeloupe are multiple resistant to insecticides (temephos, malathion and deltamethrin) as a result of a combined insecticide pressure exerted by agricultural and vector control activities [24]. Nevertheless, little is known about the resistance status of *Cx*. *quinquefasciatus* mosquitoes from Guadeloupe as the only insecticide resistance assessment for this species have been performed in 1999 and no mechanisms of resistance were investigated so far [18]. As *Cx*. *quinquefasciatus* and *Ae*. *aegypti* can share habitats and coexist in urban areas [34], it is probable that the large deployment of insecticides to control *Ae*. *aegypti* mosquitoes have also secondarily impacted the insecticide resistance levels of neighboring populations of *Cx*. *quinquefasciatus*, even if this latter species was not primarily targeted by the local vector control activities in Guadeloupe (i.e. deltamethrin spraying). In this study, we assessed the resistance levels of three *Cx*. *quinquefasciatus* populations from Guadeloupe to malathion, temephos and deltamethrin. We have also characterized for the first time resistance mechanisms in *Cx*. *quinquefasciatus* populations from Guadeloupe. Among them, the frequency of most commons target-site modifications described in *Cx*. *quinquefasciatus*: (i) the L1014F mutation in the VGSC gene, conferring resistance to pyrethroids and DDT (Dichlorodiphenyltrichloroethane insecticide) [33,35,36], and (ii) the G119S mutation in the a*ce-1* gene, conferring resistance to temephos and malathion [32]. In addition, we have determined the constitutive expression levels of five cytochrome P450s that have been shown to be involved in insecticide detoxification in *Cx*. *quinquefasciatus* [30,31,37]. Finally, we have performed association analysis between the insecticide resistance phenotypes and the resistance mechanisms explored.

## Materials and methods

### Ethics statement

This study has been approved by the internal ethics committee of the Pasteur Institute of Guadeloupe. Anubis Vega-Rúa (author of the study) provided a written consent for blood donation to artificially feed mosquitoes.

### Mosquito collection and rearing

**Fig 1:**
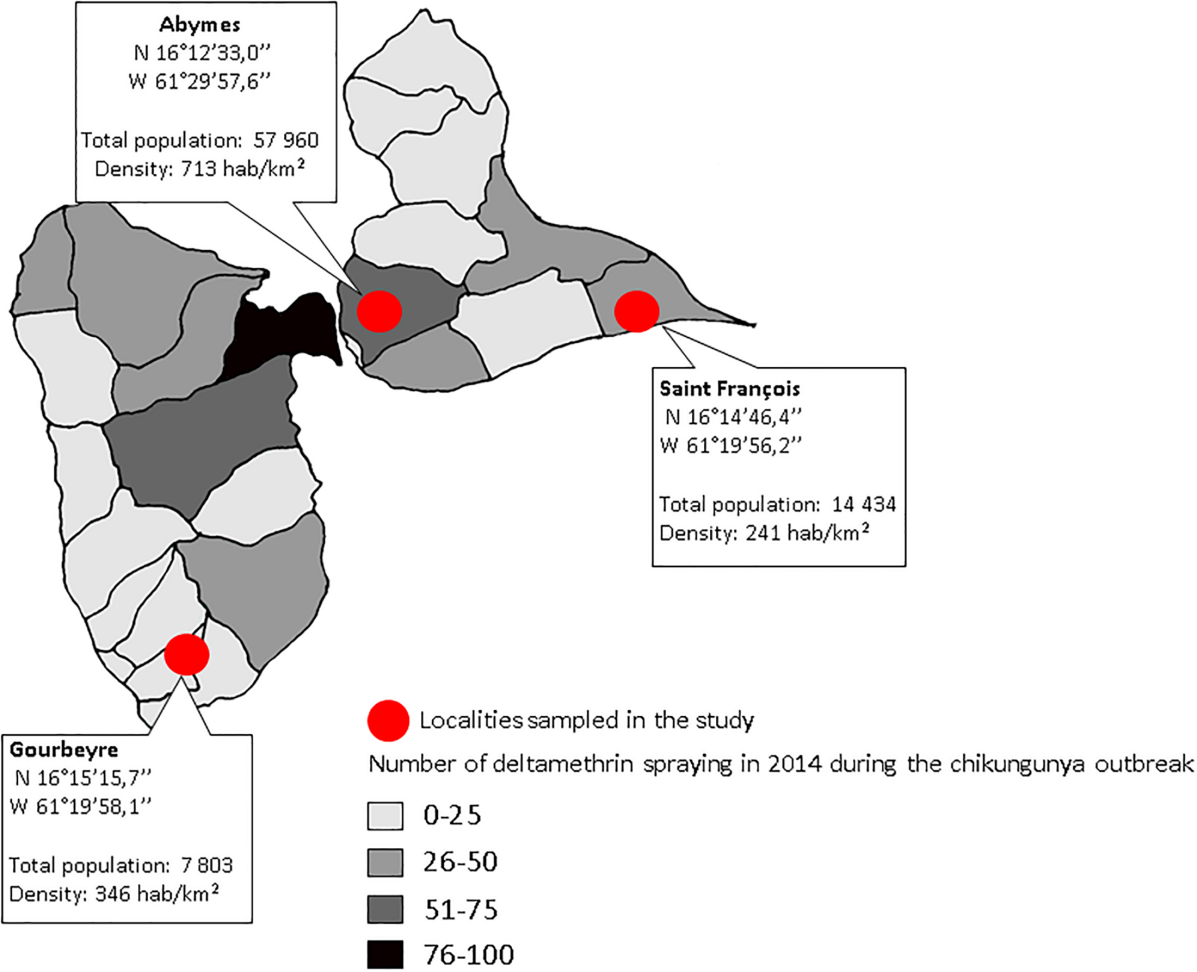
Collection sites of *Culex quinquefasciatus* populations used in the study. The red dots correspond to mosquito collection sites and a scaled shade of gray indicates different number of deltamethrin spraying conducted by municipality in Guadeloupe in 2014. Map source: drawn by the authors.

Larvae of *Cx*. *quinquefasciatus* were collected from March to April 2016 in three different locations of Guadeloupe: Gourbeyre (N 16°15’15,7”W 61°19’58,1”), Saint François (N 16°14’46,4”W 61°19’56,2”) and Abymes (N 16°12’33,0”W 61°29’57,6”). These mosquito collection sites are located in zones that often undergo a contrasted number of insecticide treatments, because of their differences in terms of human population size and density (Fig 1). Mosquito samplings were performed around private houses randomly chosen and where the residents gave their permission for the larvae collections. Larvae and pupae were then brought to the insectaries and split into 150–200 individuals per container of dechlorinated tap water supplemented with rabbit food. Adults that emerged were maintained in cages at 27°±1°C, 80% of humidity, 12h:12h light:dark cycle (inverted) and supplied with a 10% sucrose solution. Human blood from volunteers was provided to female mosquitoes using Hemotek^®^ feeding system in order to produce eggs (first generation or F_1_). The F_1_ mosquitoes were used for all the experiments and molecular biology investigations. Slab susceptible mosquito strain was graciously provided by the Institut Pasteur in Paris [38]. Slab strain was used as a reference for all insecticide susceptibility tests.

### Larval bioassays

Larval bioassays were performed using temephos and malathion insecticides following the WHO (World Health Organization) recommendations [39]. The late third and early fourth-instar larvae were used for each mosquito population. Four replicates per concentration and five to six concentrations were tested with 25 larvae per replicate and concentration. The insecticide alcohol dilutions tested were provided by WHO Universiti Sains Malaysia manufacturer. Temephos and malathion “stock” solutions received were at 156.25μg/ml and 31.25μg/ml. For testing the field mosquito samples, the concentrations were adjusted to include different percentages of survival/mortality, varying from 0% to 100%. The concentrations tested for field samples were 0.001, 0.003, 0.005, 0.007, 0.01μg/ml for temephos and 0.01, 0.025, 0.05, 0.1, 0.3μg/ml for malathion, while concentrations tested for the reference Slab strain were 0.0001, 0.0005, 0.001, 0.003, 0.005, 0.01μg/ml for temephos and 0.0075, 0.01, 0.025, 0.05, 0.1μg/ml for malathion. The results were analyzed with XLSTAT-Biomed software (XLSTAT 2015) to determine the lethal concentration for 50% (LC_50_) of the populations. Resistance ratio (RR_50_) was calculated using LC_50_ rates from *Cx*. *quinquefasciatus* field sampled populations compared with the LC_50_ rate of the susceptible Slab strain. RR_50_≤1 indicates susceptibility to the tested insecticide, while RR_50_>1 indicates insecticide resistance. The resistance levels were also ranked in 3 categories according to Mazzarri & Georghiou (1995) [40] and Bisset et al. (2011) [41] as follows: low resistance for RR_50_<5, moderate resistance for 5≤RR_50_≤10 and high resistance for RR_50_>10.

### Adult bioassays

Adult bioassays were performed following adapted WHO recommendations [42,43]. Deltamethrin impregnated papers were used at different concentrations: 0.01%, 0.025%, 0.05%, 0.08% for field samples and 0.0001%, 0.001%, 0.01%, and 0.025% for the reference Slab strain. For each mosquito population and each concentration, four replicates and two negative controls of 25 females (2–5 days old) were tested. Whatman n°1 papers were impregnated in the laboratory with deltamethrin PESTANAL^®^ (Sigma-Aldrich, Inc.) at the desired concentration in a solution containing 2/3 Acetone (Sigma-Aldrich, Inc.) and 1/3 silicon oil (VWR^®^). The impregnation was conducted following WHO procedures [42], using 2ml of insecticide solution to impregnate one 12x15cm paper that was allowed to dry overnight and stored at 4°C. Adult mosquitoes were exposed by tarsal contact for one hour to deltamethrin impregnated papers. The knock-down (after 1h exposure) and the mortality rates (after 24 hours) were recorded.

### Genotyping of target-site mutations

Genomic DNA was extracted from 30 *Cx*. *quinquefasciatus* females from each of the three populations studied using CTAB technique [44]. The extracted DNA was used to genotype L1014F *kdr* mutation of the VGSC and G119S *ace-1* mutation. The L1014F was genotyped using the double PCR-based assay described by Martinez-Torrez et al. [33] using a Dreamtaq green PCR master mix (Thermoscientific^®^) and the following PCR conditions: 5 min at 94°C followed by 40 cycles of 94°C for 30 sec, 55°C for 30 sec and 72°C for 30 sec, with a final extension of 7min at 72°C. The G119S a*ce-1* mutation was investigated using the PCR-RFLP (PCR Restriction Fragment Length Polymorphism) test developed by Weill et al. [32]. The PCR conditions were slightly modified as follows: 5 min at 94°C, 30 cycles (94°C for 30 sec, 52°C for 1 min and 72°C for 1 min) and a final extension of 7 min at 72°C. Primers used are shown in Table 1.

**Table 1:** Primers used for the screening of target-site modifications and the expression analysis of five cytochrome P450 genes in *Culex quinquefasciatus*.

Mutation/Gene	Primer name	Primer sequence 5'-3'	References
**Target-site modifications**			
L1014F -*kdr* mutation	Cgd1	GTGGAACTTCACCGACTTC	Martinez-Torres et al., 1999
	Cgd2	GCAAGGCTAAGAAAAGGTTAAG	Martinez-Torres et al., 1999
	Cgd3	CCACCGTAGTGATAGGAAATTTA	Martinez-Torres et al., 1999
	Cgd4	CCACCGTAGTGATAGGAAATTTT	Martinez-Torres et al., 1999
G119S -*ace-1* mutation	Moustdir1	CCGGGNGCSACYATGTGGAA	Weill et al., 2004
	Moustrev1	ACGATMACGTTCTCYTCCGA	Weill et al., 2004
**Cytochrome P450 Genes**			
*CYP4H34* (XM_001861335.1)	*CYP4H34*-up	CATCCAGCTGGCAAAGCACC	Hardstone et al., 2010
	*CYP4H34*-do	GACTTCTGCGCCGAGTACG	Hardstone et al., 2010
*CYP9J40* (JF501091.1)	*CYP9J40*-liu- F	ACCCGAATCCGGGCAAGTTTGAT	Liu et al., 2011
	*CYP9J40*-liu-R	AACTCCAAACGGTAAATACGCCGC	Liu et al., 2011
*CYP9J45* (XM_001855163.1)	*CYP9J45*-Gong-F	TCAGCGGTACGGAAACGATGTGAT	Gong et al., 2013
	*CYP9J45*-Gong-R	AGTCCATGTTGGTCTTCTGTCCCA	Gong et al., 2013
*CYP9M10* (JF501093.1)	*CYP9M10*-liu-F	ATGCAGACCAAGTGCTTCCTGTAC	Liu et al., 2011
	*CYP9M10*-liu-R	AACCCACTCAACGTATCCAGCGAA	Liu et al., 2011
*CYP6AA7* (JF501089.1)	*CYP6AA7*-liu-F	ATGACGCTGATTCCCGAGACTGTT	Liu et al., 2011
	*CYP6AA7*-liu-R	TTCATGGTCAAGGTCTCACCCGAA	Liu et al., 2011
60S Ribosomal protein L8 (XM_001841875.1)	quinque-*RPL8*-F	GCTGGCCGAAGGTGCGTGGT	Present study
	quinque-*RPL8*-R	TTGCGACCTGGCGGCGTTCC	Present study

Parenthesis indicates gene accession number in Genbank

### Expression of cytochrome P450 genes

RNA was extracted from three pools of 16 females for each of the three different mosquito populations using trizol/chloroform (Invitrogen, Carlsbad, CA, USA) method and cDNA was synthetized with Super Script VILO Master Mix (Invitrogen), after a DNAse treatment with DNAse I Amplification grade (Invitrogen). The PCR reaction for relative quantification was made in 15μl including 7.5μl of Sybr Green Master Mix, 0.45μl of 10μM of both primers, 3.6μl H_2_O and 3μl of cDNA at 30ng/μl. The thermocycling conditions were: 95°C for 3 min (holding stage), 50 cycles of 95°C for 15 sec and 60°C for 32 sec (cycling stage), and finally a melt curve stage. The differential expression of cytochrome P450 genes *CYP4H34*, *CYP9J40*, *CYP9J45*, *CYP9M10* and *CYP6AA7* for the three populations studied was calculated through the ΔΔCt method taking into account the PCR efficiency. The fold-change of transcripts using as the baseline the expression obtained for the Slab strain with *RPL8* endogenous gene was obtained using the DataAssist v3.01 software. Each run of real-time PCR included gene expression measurement of *RPL8* endogenous gene and the target gene in the corresponding samples. The primers used (Table 1) were taken from previous studies except for those encoding the 60S ribosomal protein L8 endogenous gene (*RPL8*) that were specially designed for the present study using the NCBI Primer-Blast tool online.

### Statistical analysis

Statistical analysis were performed with XLSTAT 2015 (Addinsoft, France) software. XLSTAT was used (i) to perform log-probit logistical regression to investigate dose-effect relationship regarding insecticide tests data, (ii) to compare adult KD/mortality (Deltamethrin) and larval mortality (temephos and malathion) between mosquito populations via ANOVA and Kruskal-Wallis tests, (iii) to perform pairwise comparisons (Students t-test) of gene expression ratios between Slab strain and the field populations and (iv) to analyze the association between the obtained variables via a Principal Component Analysis (PCA). The frequency data from the *ace-1* and VGSC genes were analyzed using the Genepop software [45] to check Hardy-Weinberg equilibrium for each mosquito population. The overall significance of multiple tests was estimated with Fisher’s combined probability test and the p-value corrected with the sequential Bonferroni procedure [46].

## Results

### Insecticide resistance profile of *Cx*. *quinquefasciatus* for temephos, malathion, and deltamethrin

Larval and adult bioassays conducted revealed multiple resistances in *Cx*. *quinquefasciatus* populations from Guadeloupe (Table 2). The lowest resistance ratios (RR_50_) were observed for temephos (RR_50_ ranged between 1–2), indicating low resistance for *Cx*. *quinquefasciatus* populations except for mosquitoes from Saint François which are still susceptible for this insecticide (RR_50_ = 1). This latter population also displayed significant higher mortality (Table 3) than the populations from Abymes and Gourbeyre when exposed to this insecticide (Kruskal-Wallis H-test: p<0.0001). Concerning malathion, mosquito populations from Abymes and Gourbeyre displayed moderate resistance with RR_50_ of 5.38 and 9.75 respectively, while the population from Saint François was found to be still susceptible to this insecticide (RR_50_ = 0.75). In addition, the mortality of three populations was statistically different for all the malathion doses except 0.3μg/ml (Kruskal-Wallis H-test: p<0.0001). The higher mortality after exposure to malathion was displayed by the population of Saint François, followed by Abymes and Gourbeyre (Table 3). All *Cx*. *quinquefasciatus* populations were shown to be highly resistant to deltamethrin with RR_50_ varying from 20 (Saint François) to 29 (Abymes). No significant differences have been observed in mortality between mosquito populations, but the *Cx*. *quinquefasciatus* population from Abymes displayed a significant lower KD rate when compared to populations from Saint François and Gourbeyre (Kruskal-Wallis H-test: p<0.0001). Overall, Abymes was the most resistant *Cx*. *quinquefasciatus* population of Guadeloupe (except for malathion), while Saint François was the most susceptible.

**Table 2:** Resistance status of *Culex quinquefasciatus* populations from Guadeloupe to deltamethrin (adults), malathion (larvae) and temephos (larvae).

Population/strain	Abymes	Saint Francois	Gourbeyre	Slab
**Deltamethrin**				
LC_50_ (±95% CI)	0.029 (0.023–0.034)	0.020(0.015–0.023)	0.026 (0.021–0.031)	0.001(-)
RR_50_	29 (23–34)	20 (15–23)	26 (21–31)	-
**Temephos**				
LC_50_ (±95% CI)	0.006(0.005–0.006)	0.003 (-)	0.005(0.004–0.006)	0.003 (-)
RR_50_	2 (1.6–2)	1 (-)	1.67 (1.33–2)	-
**Malathion**				
LC_50_ (±95% CI)	0.129(0.113–0.150)	0.018(0.010–0.026)	0.234(0.207–0.268)	0.024(0.022–0.026)
RR_50_	5.38 (4.71–6.25)	0.75 (0.42–1.08)	9.75 (8.63–11.16)	-

LC_50_: Lethal concentration for 50% of individuals. Resistance Ratios RR_50_ = LC_50_ field samples/ LC_50_ Slab strain.

**Table 3:** Mortality and Knock-down rates of *Culex quinquefasciatus* populations from Guadeloupe exposed to deltamethrin (adults), malathion (larvae) and temephos (larvae).

Population		Abymes		Saint François		Gourbeyre	
Insecticide	Dose (μg/ml)*	KD(%)	Mortality(%)	KD(%)	Mortality (%)	KD(%)	Mortality(%)
Deltamethrin	0.01%	21.6	24.7	49.5	36.1	54.5	26.3
	0.025%	52.6	39.1	58.7	54.3	77.1	45.7
	0.05%	75.5	72.4	88.5	84.7	74.7	74.4
	0.08%	87.7	75.5	100	85.4	96.78	75.7
Temephos	0.001	-	0	-	10.7	-	0
	0.003	-	17	-	23	-	23.3
	0.005	-	49	-	98	-	51.3
	0.007	-	60	-	100	-	96.7
	0.01	-	90	-	100	-	100
Malathion	0.01	-	0	-	38	-	0
	0.025	-	1	-	55	-	0
	0.05	-	18	-	70	-	1
	0.1	-	35	-	73	-	6.5
	0.3	-	84	-	79	-	64.5

*Doses are given in μg/ml except when otherwise noted.

KD: Percentage of knock-down mosquitoes after one hour of deltamethrin exposure. Mortality: Percentage of dead mosquitoes 24 h after insecticide exposure.

### Genotyping of L1014F *kdr* and G119S *ace-1* mutations

**Fig 2:**
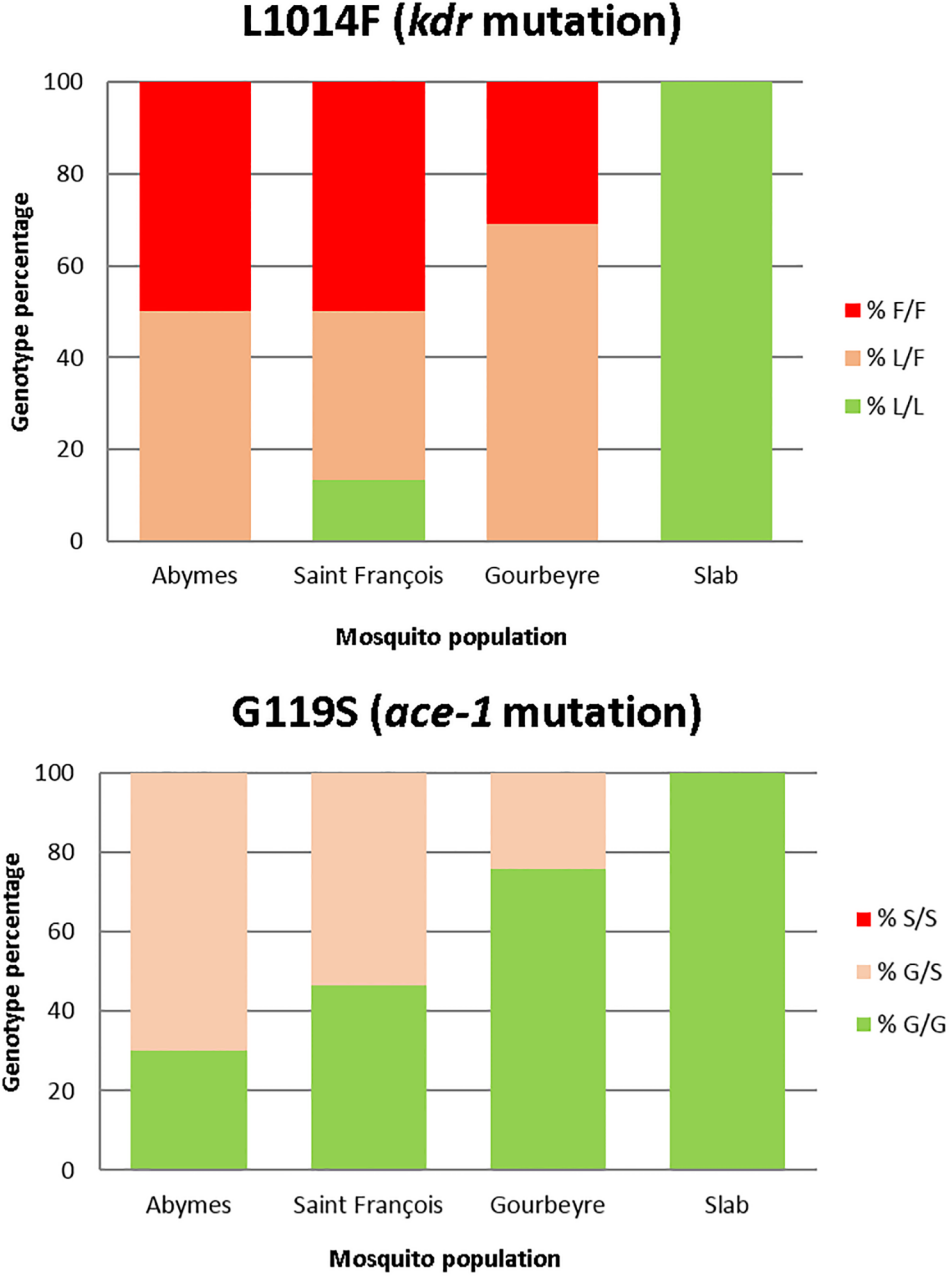
Genotype proportions of the mutations L1014F (*kdr*) and G119S (*ace-1*) in the three *Culex quinquefasciatus* populations studied and the Slab reference strain. L/L: wild homozygote genotype (susceptible); L/F: L1014F heterozygote genotype; F/F: L1014F mutant homozygote (resistant); G/G: G119S wild homozygote genotype (susceptible); G/S: G119S heterozygote genotype; S/S: G119S mutant homozygote (resistant).

A high frequency of the L1014F *kdr* mutation was detected in all the three *Cx*. *quinquefasciatus* populations from Guadeloupe. Whatever the mosquito population, the frequency of the mutant and resistant allele (F) was higher than the wild “susceptible” allele (L): 0.66 for Gourbeyre, 0.68 for Saint François and 0.75 for Abymes. The resistant homozygote genotype (F/F) was found more frequently in the populations Abymes and Saint François (50% of individuals) than in Gourbeyre (32% of individuals) (Fig 2). The highest proportion of heterozygote genotype (L/F) was found in Gourbeyre (69%), while the lowest proportion was observed in mosquitoes from Saint François (37%). The wild homozygote susceptible genotype (L/L) was only found in the *Cx*. *quinquefasciatus* population from Saint François. Significant deviations from Hardy-Weinberg expectations (Hardy-Weinberg exact test: p = 0.02) were detected for this locus due to a significant excess of heterozygotes in the Gourbeyre population (Hardy-Weinberg exact test for alleles: p = 0.0057).

Regarding the G119S *ace-1* mutation, the frequencies of the resistant allele (S) were lower than those of the wild “susceptible” allele (G) in all populations: 0.35 for Abymes, 0.27 for Saint François and 0.12 for Gourbeyre. In addition, no homozygote resistant genotype (S/S) was detected in any of the populations (Fig 2). Nevertheless, the proportion of individuals with the heterozygote genotype harboring the resistant mutation (G/S) was very high in the population Abymes (70%), followed by Saint François (53.3%). The majority of individuals from Gourbeyre (75.9%) still harbor the wild homozygote susceptible (G/G) genotype. Significant deviations from Hardy-Weinberg expectations (Hardy-Weinberg exact test: p = 0.014) were detected for this locus only in the Abymes population due to an excess of hererozygotes (Hardy-Weinberg exact test for alleles: p = 0.0038). Neither *kdr* nor *ace-1* mutation was found in the susceptible Slab strain.

### Transcriptional profiling of selected cytochrome P450s expression levels in *Cx*. *quinquefasciatus*

**Fig 3:**
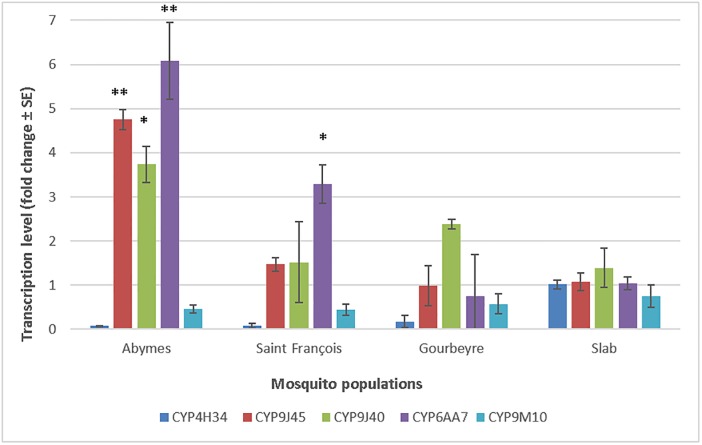
Constitutive transcription levels of the cytochrome P450 monooxygenase genes *CYP4H34*, *CYP9J45*, *CYP9J40*, *CYP6AA7* and *CYP9M10* in three *Culex quinquefasciatus* populations from Guadeloupe compared to the susceptible Slab strain. The transcription ratios obtained from real-time quantitative PCR were normalized with the housekeeping gene *RPL8* and shown as mean value (±SE) of three independent biological replicates. Asterisks indicate genes over-transcribed with transcription ratio fold ≥2 and with significant P-values: p<0.05 (*) and p<0.01 (**).

The transcription profiles of the five candidate detoxification genes potentially involved in metabolic resistance to insecticides in the three *Cx*. *quinquefasciatus* populations from Guadeloupe were compared to those of the susceptible Slab strain (Fig 3). Genes with transcription ratio (TR)≥2 and P-value≤0.05 (according to the T-student test) compared to the Slab strain were considered as significantly over-transcribed. Only the *CYP9J45*, *CYP9J40* and *CYP6AA7* were significantly over-transcribed. The *CYP6AA7* was over-expressed in mosquitoes from Abymes (TR = 6.08; p = 0.0049) and Saint François (TR = 3.29; p = 0.05), while *CYP9J45* and *CYP9J40* were over-transcribed only in Abymes (*CYP9J45*: TR = 4.75; p = 0.0013 and *CYP9J40*: TR = 3.74; p = 0.036). The *CYP9M10* and *CYP4H34* were not over-transcribed in any population when compared to the Slab strain (TR<2).

### Association between insecticide resistance levels, target-site mutations and expression levels of detoxification enzymes

**Fig 4:**
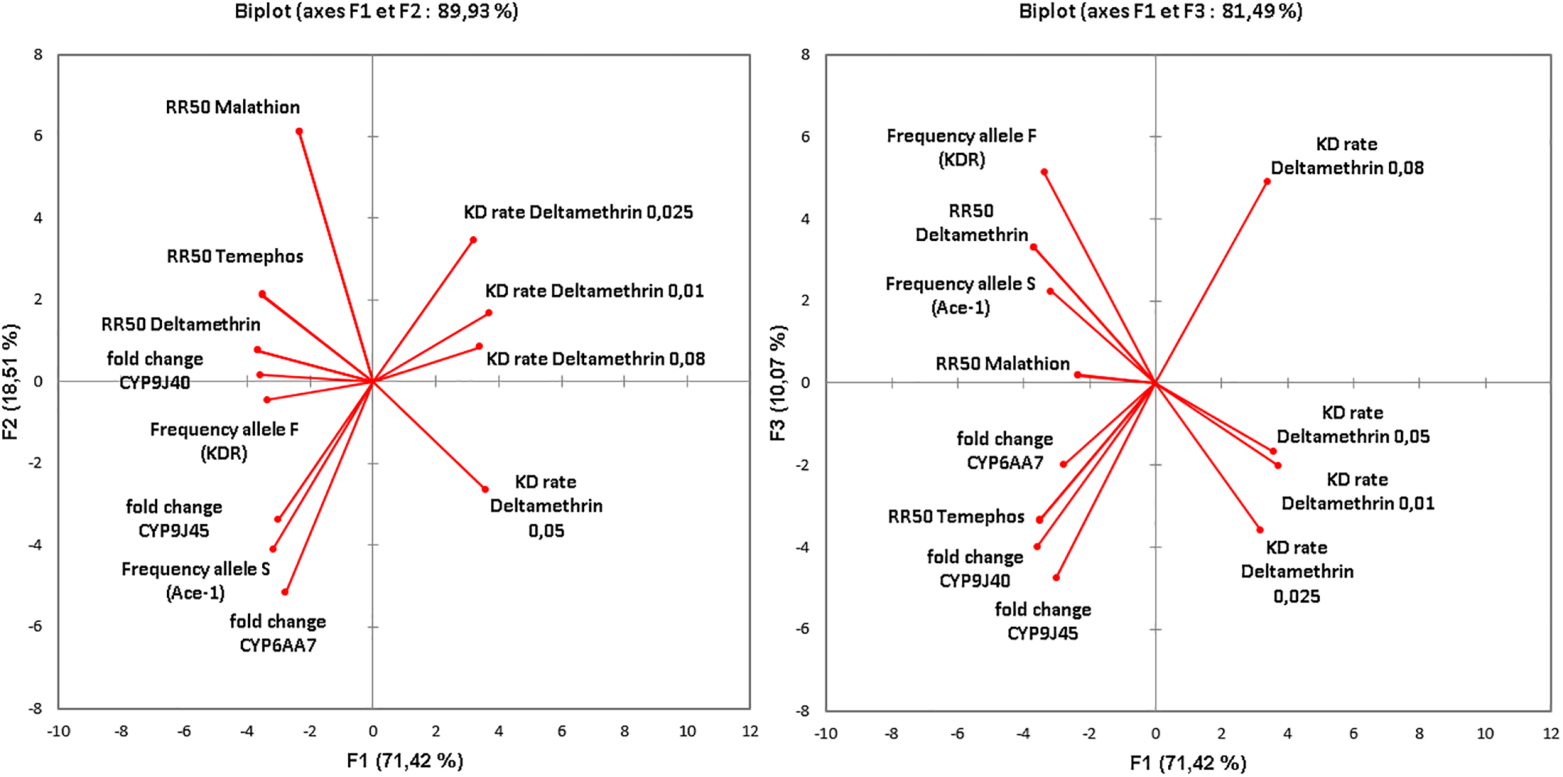
Principal component analysis for 12 variables including (i) larval and adult resistance levels (RR_50_) regarding temephos malathion and deltamethrin, (ii) deltamethrin knock-down rates for each concentration used, (iii) adult transcription ratios of cytochrome *CYP6AA7*, *CYP9J40* and *CYP9J45* genes, and (iv) the resistant allele frequencies for *kdr* and *ace-1* mutations for the three *Culex quinquefasciatus* populations tested.

A Principal Component Analysis (PCA) was performed for all *Cx*. *quinquefasciatus* populations using 12 variables such as (i) larval and adult resistance levels (RR_50_) for temephos, malathion and deltamethrin, (ii) deltamethrin knock-down rates for each concentration used, (iii) adult transcription ratios of cytochrome P450s *CYP6AA7*, *CYP9J40* and *CYP9J45* and finally (iv) the allelic frequencies of *kdr* and *ace-1* mutations. The PCA results are summarized in the Fig 4.

The two first PCA axes represented 89.93% of the starting information with respectively 71.42% and 18.51%, while the third axis represented only 10.07%. Positive but no significant correlations were found between the resistance ratios of adult mosquitoes for deltamethrin and those obtained with mosquito larvae for temephos (Correlation: r(132) = 0.78, p>0.05) and malathion (Correlation: r(132) = 0.65, p>0.05). Resistance to deltamethrin was strongly correlated with the frequency of the resistant *kdr* allele (F) (Correlation: r(132) = 0.97, p = 0.031), while resistance to temephos was found to be correlated to the expression fold change of *CYP9J40* (Correlation: r(132) = 0.965, p = 0.035). The knock-down rates found for deltamethrin displayed strong negative correlations with the resistant allele frequencies of *ace-1* mutation (Correlation: r(132) = -0.99, p = 0.013), with the expression fold change of *CYP9J40* (Correlation: r(132) = -0.98, p = 0.013), with deltamethrin resistance (Correlation: r(132) = -0.96, p = 0.043) and to a lesser extent with the resistant allele frequency of *kdr* mutation (Correlation: r(132) = -0.94, p = 0.061). No significant associations were detected between the expression fold levels of *CYP9J45* and *CYP6AA7* and any of the tested variables.

## Discussion

In this study we investigated the resistance status of *Cx*. *quinquefasciatus* mosquitoes from Guadeloupe against malathion, temephos and deltamethrin: three insecticides that have been historically used for chemical control of the mosquito vector *Ae*. *aegypti* in the island. Malathion and temephos were respectively used in Guadeloupe for adult and larval vector control from the late 1960s to 2009–2010, while deltamethrin has been used from the early 1980s until the present day (Pasteur Institute archives). Bioassays performed on the three *Cx*. *quinquefasciatus* populations from Guadeloupe revealed high resistance levels to deltamethrin (20<RR_50_<29), moderate resistance to malathion (0.75<RR_50_<9.75) and a low resistance to temephos (1<RR_50_<2). *Cx*. *quinquefasciatus* populations from Guadeloupe have been already found to be resistant to deltamethrin since the late 1990s [18], but the resistance to malathion had never been described for this mosquito species in Guadeloupe until now. However, the resistance to malathion has already been reported for *Cx*. *quinquefasciatus* populations from Latin America with RR_50_ values sometimes up to 200 [34]. The differences on the resistance levels observed for these two adulticides in the studied populations from Guadeloupe are in agreement with a more recent and higher deltamethrin selective pressure, as this insecticide is still used for vector control activities in the island (last massive spraying in 2014 during the chikungunya outbreak), while malathion has not been not used since 2010 because the insecticide was withdrawn from the list of vector control insecticides [25]. The resistance observed in *Cx*. *quinquefasciatus* mosquitoes regarding these two insecticides is possibly due to the lack of specificity of indoor and outdoor spraying used for *Ae*. *aegypti* control in Guadeloupe. This “secondary resistance” developed by a non-target organism has been often reported for different insect species [47]. In the case of *Cx*. *quinquefasciatus*, this finding is not surprising as this mosquito is a domestic species highly adapted to the urban environment that often co-exists with *Ae*. *aegypti* (especially the adult stages) in human habitations [48]. Interestingly, *Cx*. *quinquefasciatus* populations presented more heterogeneous resistance levels (0.75<RR_50_<9.75) when compared to *Ae*. *aegypti* (1.68<RR_50_<4.39) [24], which could be possibly explained by contrasted or unequal levels of co-habitation with *Ae*. *aegypti* in the sampled zones (i.e. greater *Cx*. *quinquefasciatus—Ae*. *aegypti* co-habitation in zones were breeding-sites are more scarce or insufficient).

The lowest resistance levels were obtained for temephos. A slight resistance was observed in populations from Abymes (RR_50_ = 2) and Gourbeyre (RR_50_ = 1.67), while the population from Saint François was susceptible to this insecticide (RR_50_ = 1). These results are in agreement with a study conducted in Brazil were RR_50_ values reported were low (RR_50_<2) [49]. As previously mentioned, temephos has been used in Guadeloupe for larval vector control for about 40 years (Pasteur Institute archives) and the first resistance to this insecticide in the island was reported as early as 1984 for *Ae*. *aegypti* mosquitoes (Pasteur Institute archives). In 1999, resistance to temephos was detected for the first time in *Cx*. *quinquefasciatus* mosquitoes from Guadeloupe with resistance ratios comprised between 4.47 and 32.58 [18]. Despite the difficulty to compare insecticide resistance assessments that were performed with 19 years of difference using different mosquito populations from Guadeloupe, the resistance levels obtained in the present study are consistent with the withdrawal of temephos for the vector control activities [25] and thus, with a lower temephos-associated selective pressure. In addition, temephos treatments in the last decades were principally used for *Ae*. *aegypti* breeding-sites and not for those infested with *Culex* spp, which may have also contributed to a faster decrease of temephos resistance levels in *Cx*. *quinquefasciatus* populations.

Concerning the insecticide resistance mechanisms, the *kdr* mutation L1014F was more frequent in *Cx*. *quinquefasciatus* populations than the *ace-1* mutation G119S (Fig 2). Homozygote resistant individuals (F/F genotype) were observed for the *kdr* mutation only, and almost no homozygote susceptible genotypes (L/L) have been found among the analyzed mosquitoes, suggesting a higher selective pressure for this particular mutation than for *ace-1*. The L1014F *kdr* mutation has been shown to confer resistance to pyrethroids in *Cx*. *quinquefasciatus* [33,35], which can explain the high resistance values obtained for the adulticide deltamethrin (still used in Guadeloupe) for all the studied populations (RR_50_≥20). This statement is also supported by the PCA analysis that revealed strong positive correlations between deltamethrin resistance and the frequency of the resistant *kdr* allele [F] (Correlation: r(132) = 0.97, p = 0.031). The highest deltamethrin resistance and the highest frequency of L1014F *kdr* mutation were both found in the Abymes population, suggesting that a higher deltamethrin-selective pressure has been exerted in this population for decades. The spraying conducted during the chikungunya outbreak in 2014 reflects this issue, as the number of deltamethrin spraying in Abymes was 53 while in Gourbeyre only 5 insecticide spraying events have been recorded in the same period (Fig 1) (see Annex 8 in [50]). The G119S *ace-1* mutation was less frequent in the populations when compared to L1014F, but it was found in all *Cx*. *quinquefasciatus* populations as well, which could explain the moderate malathion resistance and the low temephos resistance observed in this study. As explained above, the G119S *ace-1* mutation has shown to confer resistance to organophosphorous insecticides [32,51] and has also been associated with malathion resistance in *Cx*. *quinquefasciatus* [52]. Malathion spraying conducted in the past decades and before 2010 to target adult mosquito stages could have acted as the main source of selective pressure for this target-site mutation in *Cx*. *quinquefasciatus* populations, as the highest frequency of heterozygote individuals for *ace-1* mutation harboring the resistant allele for this gene was found in Abymes, a locality considered as a transmission “hot spot” for arboviruses that has been historically more treated with insecticides when compared to Saint François and Gourbeyre (Figs 1 and 2) [50]. However, it is possible that other mechanisms non-explored in this study such as malathion detoxification by carboxylesterase enzymes [28,53–55] could have been involved in malathion resistance, as Gourbeyre population displayed the highest resistance to malathion and the lowest frequencies of the resistant allele for the *ace-1* mutation. Taken together, these results suggest an association between the resistance levels observed in *Cx*. *quinquefasciatus* for the tested insecticides, the frequency of both target-site mutations studied, and the insecticide selective pressures exerted by the past and present vector control activities (Table 2, Figs 1 and 2).

Resistance mechanisms associated to the insecticide detoxification by cytochrome P450s have also been explored for the first time in *Cx*. *quinquefasciatus* populations from Guadeloupe. Constitutive over expression of P450 genes like *CYP6AA7*, *CYP9J40*, *CYP9J45*, *CYP4H34* and *CYP9M10* has been linked to the development of resistance to pyrethroids (i.e. permethrin, deltamethrin) in *Cx*. *quinquefasciatus* [30,56]. Among the five cytochrome P450s studied, only three have found to be over-transcribed in the local *Cx*. *quinquefasciatus* populations when compared to the susceptible Slab strain: *CYP9J45*, *CYP9J40* and *CYP6AA7*. Again, the mosquito population “Abymes” had the highest number of over-transcribed cytochromes with the highest transcription levels, which is in agreement with the important resistance levels to deltamethrin observed for this population. In addition, the Principal Component Analysis revealed significant negative correlations between the expression fold change of cytochrome P450 gene *CYP9J40* and the deltamethrin-induced knock-down rates (Correlation: r(132) = -0.98, p = 0.013). The *CYP9M10* and *CYP4H34* were not over-transcribed in any tested population, which can be explained by the use of adult mosquitoes for the experiments for these two genes. Indeed, several studies have found that the constitutive over-expression of *CYP9M10* and *CYP4H34* genes is very high in the 4^th^-larval stage but subsequently drops in pupae and adult mosquitoes [30,56]. Nevertheless, further studies should be conducted to compare the constitutive expression of *CYP9M10* and *CYP4H34* genes in larvae and adult mosquitoes to verify if the same expression decrease from larvae to adult is also observed in field-collected mosquitoes. In the same way, the over-expression by insecticide induction of the studied cytochromes and other metabolic enzymes should be also deeply investigated to better characterize their role in the insecticide resistance of *Cx*. *quinquefasciatus* [31].

## Conclusions

*Cx*. *quinquefasciatus* mosquitoes from Guadeloupe are multiple resistant to insecticides even if this mosquito species is not the main target of the vector control activities in the region. The studied populations displayed high resistance to deltamethrin, moderate resistance to malathion and were susceptible-to-slightly resistant to temephos. The resistance levels observed in *Cx*. *quinquefasciatus* populations seem to be associated with the *kdr* and *ace-1* mutations, as well as with the overexpression of cytochrome P450 genes *CYP9J40 CYP6AA7*, and *CYP9J45*. The population of Abymes is the better example of the effects of insecticide selective pressure in *Cx*. *quinquefasciatus* mosquitoes, as this population has historically been prioritized for the insecticide treatments, which had led to the appearance and the selection of multiple resistances and the associated mechanisms. Indeed, the Abymes mosquito population had the highest frequency of both *kdr* and *ace-1* mutations, and the highest constitutive expression levels of the studied cytochrome P450s. *Cx*. *quinquefasciatus* mosquitoes are resistant to the only chemical insecticide that is still used in vector control strategies locally. Thus, the challenge is to manage this resistance in order to prevent the emergence of *Cx*. *quinquefasciatus*-borne diseases in Guadeloupe.
